# The mediating role of empathy between nature connectedness and social–emotional competence among upper-grade Yi ethnic minority pupils in rural Southwest China

**DOI:** 10.3389/fpsyg.2026.1694397

**Published:** 2026-01-20

**Authors:** Junchao Yuan, Gangqin Chen, Yuyang Chen, Tao Liu, Yao Xiao, Lingjun Yu, Hongye Geng

**Affiliations:** 1 School of Teacher Education, Xichang University, Xichang, Sichuan, China; 2 Henan Primary School, Weihai, Shandong Province, China

**Keywords:** cross-sectional study, empathy, nature connectedness, rural education, social–emotional competence, Yi ethnic children

## Abstract

**Background:**

Rural ethnic minority children in Southwest China face unique developmental challenges, particularly regarding social–emotional competence (SEC). While environmental psychology suggests nature connectedness (NC) promotes socio-emotional development, its mechanisms within culturally distinct groups like the Yi ethnic minority remain unexplored. This study aims to explore the relationship between NC and SEC and examine evidence for the mediating role of empathy among upper-grade Yi pupils.

**Methods:**

A cross-sectional survey was conducted with 647 Yi ethnic pupils (Grades 4–6) from rural Liangshan, China. Participants completed Chinese versions that have demonstrated acceptable psychometric properties of the Nature Connectedness Scale, the Delaware Social–Emotional Competence Scale, and the Basic Empathy Scale. Data analysis employed Pearson correlations, t-tests, ANOVA, and Hayes’ PROCESS macro (Model 4, 5,000 bootstraps) with gender as a covariate.

**Results:**

NC significantly positively predicted SEC (*β* = 0.28, *p* < 0.001). NC significantly positively predicted empathy (*β* = 0.20, *p* < 0.001). Empathy significantly positively predicted SEC (*β* = 0.27, *p* < 0.001). Empathy showed a partial mediating effect in the NC-SEC relationship (indirect effect = 0.04, 95% CI[0.02, 0.07]), accounting for 18.98% of the total effect. Significant gender differences emerged, with girls scoring higher on NC, empathy, and SEC (*p* < 0.001). No significant grade differences were found.

**Conclusion:**

This study provides evidence supporting empathy as a significant partial mediator linking nature connectedness to social–emotional competence among rural Yi children. Findings provide empirical support for the pathway within a culturally distinct context, highlighting the synergistic potential of leveraging ethnic ecological-cultural resources (e.g., nature-based rituals, traditional practices) for fostering socio-emotional development in marginalized populations. Future research should utilize longitudinal designs and incorporate multi-informant data.

## 1 Introduction

Amidst accelerating global urbanization, the developmental challenges facing rural children have garnered increasing attention, particularly in multi-ethnic regions of Southwest China experiencing unprecedented sociocultural transformations and ecological pressures. Southwest China, a convergence zone of rich biodiversity and cultural diversity, serves as the upper ecological barrier for major rivers like the Yangtze and Pearl Rivers and is home to dozens of indigenous ethnic minorities, including the Yi, Tibetan, and Miao peoples. Despite its vast territory and ecological richness, the region faces pervasive challenges including lagging economic development, structural shortages in educational resources, and weakened intergenerational transmission of traditional culture (Zheng and Liao, 2019). The healthy development of rural children, especially ethnic minority children, is crucial not only for educational equity and social justice but also profoundly impacts regional sustainable development and ethnic unity. Within this context, the developmental disparities experienced by rural ethnic minority children have become a focal point for China’s rural revitalization and national unity initiatives. Among the factors influencing child development, Social and Emotional Competence (SEC)—encompassing the cognitive, emotional, and behavioral capacities to understand and manage one’s own and others’ emotions, establish and maintain positive relationships, make responsible decisions, and constructively handle challenges [CASEL (Collaborative for Academic, Social, and Emotional Learning), 2023; Zins et al., 2004]—is recognized as a core component of essential competencies. UNICEF (2022) highlights that globally, children in marginalized regions exhibit significant lags in developmental indices, with insufficient cultivation of SEC being a key bottleneck to unlocking their potential. Research indicates that higher levels of SEC are significantly positively associated with children’s academic achievement, mental health, social adaptation, and future life satisfaction (Durlak et al., 2011; Wigelsworth et al., 2016; Taylor et al., 2017). Given the complex ecological and cultural constraints on SEC development among Yi children in rural Southwest China—particularly upper-grade primary pupils navigating the critical transition to adolescence—exploring its influencing factors and facilitating mechanisms holds urgent practical significance.

For Yi children growing up in rural Southwest China, SEC development occurs within a specific ecological-cultural context. The Yi are one of the larger ethnic minorities in Southwest China, possessing unique language, a writing system (e.g., standardized Yi script), religious rituals (e.g., Bimo culture), and a cosmology deeply rooted in nature (Liangshan Yi Autonomous Prefecture Local Chronicles Compilation Committee, 2011). However, the upbringing environment of rural Yi children embodies seemingly contradictory yet intrinsically unified characteristics: relative scarcity of material resources and educational opportunities (Wang, 2019) juxtaposed with unique ecological-cultural resources—the deep intertwining of millennia-old Yi civilization with the Hengduan Mountain ecosystem. Yi culture, grounded in the philosophical foundation of animism, constructs a cognitive framework of a “human-nature-ancestral spirits” trinity through creation epics recorded in the Bimo scriptures of the Nuosu branch (e.g., the Leyte epic), seasonal rituals based on mountain agriculture (e.g., insect-repelling blessings during the Torch Festival), and worship systems for sacred mountains (e.g., Luojishan) and sacred trees (e.g., Yunnan hackberry; Liu and Cihuo, 2022). This traditional culture embodies profound reverence and interdependence with natural elements like mountains, forests, and flora/fauna. This cultural heritage shapes Yi children’s nature connectedness—the psychological trait reflecting an individual’s sense of belonging and unity with nature at the affective, cognitive, and behavioral levels (Mayer and Frantz, 2004)—with distinct cultural situatedness.

Recent environmental and positive psychology research suggests nature connectedness is not only a strong predictor of pro-environmental behavior and subjective well-being (Nisbet et al., 2009; Mackay and Schmitt, 2019), but also promotes mental health (Capaldi et al., 2014; Pritchard et al., 2020) and prosocial development (Weinstein et al., 2009; Goldy and Piff, 2020; Pirchio et al., 2021). It may also indirectly enhance social development by boosting psychological resilience and facilitating social interaction (Kuo et al., 2019). Theoretical frameworks such as Attention Restoration Theory (ART; Kaplan, 1995) posit that natural environments possess inherent properties that can replenish depleted cognitive resources, such as directed attention. This restorative function is considered a key mechanism through which engagement with nature may support broader cognitive and emotional regulation capacities. For rural Yi children whose environment retains more natural elements and whose cultural traditions emphasize human-nature harmony, their patterns of interaction with nature are highly culturally embedded. Consequently, their level of nature connectedness and its potential positive effects may hold unique significance.

Nevertheless, existing nature connectedness research exhibits a “triple limitation”: (1) heavy reliance on Euro-American urban middle-class samples, neglecting cultural diversity; (2) scant attention to middle childhood (9–12 years), a sensitive window for SEC development; and (3) insufficient exploration of underlying psychological mechanisms. Studies on children’s nature connectedness predominantly focus on urban children in developed Western countries, examining links to pro-environmental behavior, subjective well-being, etc. (Bruni et al., 2008; Arbuthnott, 2023; Yu and Reiss, 2025). Research is notably scarce on the connotations, levels, and relationships with key developmental competencies—particularly SEC—among rural children growing up in the ethnic cultural context of Southwest China. Traditional Yi culture, such as animistic beliefs and totemic worship of specific natural entities (e.g., Rhododendron delavayi, eagles), may profoundly shape Yi children’s cognitive, affective, and behavioral orientations toward nature. This culturally rooted nature connectedness could exert unique influences on their psychosocial development. For instance, play, labor in nature, and traditional rituals related to nature (e.g., seasonal/nature-themed celebrations) may provide crucial contexts for learning emotional regulation, understanding social rules, and cultivating empathy and collaborative spirit.

A key potential mediating mechanism underlying this relationship is empathy. Empathy is the ability to recognize and understand others’ emotional states (cognitive empathy) and generate corresponding emotional responses (affective empathy; Davis, 1983; Decety and Cowell, 2014). As a cornerstone of SEC, empathy is essential for children to build positive peer relationships, reduce aggressive behavior, develop prosociality, and make moral judgments (Eisenberg et al., 2014; van Lissa et al., 2016; Sesso et al., 2021). Consistent with Attention Restoration Theory (Kaplan, 1995), empirical research indicates that exposure to nature can support children’s cognitive functioning. For instance, Patwary et al. (2024) found that such exposure enhances cognitive flexibility and attentional restoration. Furthermore, nature-based activities may create more opportunities for social interaction (e.g., cooperative exploration), which could foster empathy (Di Fabio and Kenny, 2021; Wang et al., 2023). Some research suggests that deeper nature connectedness (transcending mere exposure to encompass belongingness and identification) may enhance empathy (especially affective empathy) by intensifying children’s emotional attention and empathetic understanding toward “others,” including plants and animals (Lumber et al., 2017; Lengieza and Aviste, 2025). For example, a child’s compassion for an injured tree might form the emotional foundation for empathizing with an injured peer (Jiao and Luo, 2022).

Notably, Yi children’s everyday practices within traditional communities—such as accompanying elders to gather medicinal herbs (e.g., identifying Poria cocos), participating in collective herding, or performing nature-themed songs and dances during “Kuxi” (New Year) festivities—offer embodied arenas for socio-emotional learning. These cultural practices may connect nature experiences with social competence by activating a key psychological mechanism: empathy. For Yi rural children, nature connectedness, rooted in their ethnic culture and daily practices, may enhance their empathic capacity toward others (including non-human life), subsequently promoting broader SEC development. Cultivating empathy for natural life (e.g., an injured lamb) may generalize to empathetic care for human peers, catalyzing overall SEC development. However, this theoretical pathway lacks empirical testing within specific cultural groups (e.g., Yi upper-grade primary pupils). A recent review also emphasized that exploring specific mediating pathways, such as empathy and perspective-taking, between nature connectedness and child development, particularly social development, is an urgent direction for future research (Arola et al., 2023).

Therefore, this study focuses on upper-grade (Grades 4–6) primary pupils in rural Yi communities of Southwest China. This age group is undergoing a critical transition from childhood to adolescence, characterized by rapid development of abstract cognitive abilities and social needs, representing a crucial window for shaping SEC (Siegel, 2012). Simultaneously, upper-grade pupils demonstrate sufficient capacity to understand and report their emotional experiences and feelings toward nature (Choe and Sheffield, 2025). Based on previous research, this study examines the following hypotheses:

*Hypothesis H*1: nature connectedness significantly predicts SEC.

*Hypothesis H*2: nature connectedness significantly predicts empathy.

*Hypothesis H*3: empathy significantly predicts SEC.

*Hypothesis H*4: empathy significantly mediates the relationship between nature connectedness and SEC.

**Figure 1:**
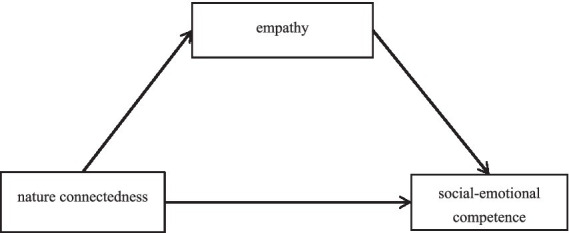
Hypothetical mediation model.

Integrating perspectives from environmental psychology (nature connectedness), developmental psychology (child empathy), educational psychology (SEC), and cultural anthropology (Yi culture), this empirical study explores these relationships within a distinct ethnic minority child group. It aims to elucidate the psychological mechanism—the potential mediating role of empathy (see Figure 1)—through which nature connectedness promotes socio-emotional development. This contributes to expanding and deepening our understanding of how natural environments influence children’s social development through specific psychological processes, providing scientific evidence for the concept of “harmonious coexistence between humans and nature” in the field of child psychological development. Practically, the findings offer insights for reforming and developing rural education in Southwest ethnic regions and provide empirical reference for educational support strategies under China’s rural revitalization strategy and the goal of fostering a strong sense of community for the Chinese nation in ethnic areas.

## 2 Method

### 2.1 Research procedure and participants

A convenience sampling method was employed to select two rural primary schools (A and B) in the Liangshan Yi Autonomous Prefecture, the largest Yi ethnic enclave in Southwest China. Using a cluster sampling approach, Yi ethnic minority students in grades 4 to 6 were selected as participants. Trained research assistants, assisted by rural primary school teachers, administered paper-based questionnaires in classrooms on May 12, 2025. The survey administration followed a standardized protocol and took approximately 20 min. Informed consent was obtained from guardians prior to participation. Ethical approval for the study was granted by the Xichang University Ethics Committee (Approval No. xcc-jsjy-psy-0501).

A total of 668 paper questionnaires were distributed, with 647 valid questionnaires returned, yielding an effective response rate of 96.86%. The sample comprised 347 boys (53.63%) and 300 girls (46.37%). Grade distribution was as follows: 270 students in Grade 4 (41.73%), 138 in Grade 5 (21.33%), and 239 in Grade 6 (36.94%). School A had 312 participants (48.22%) and School B had 335 participants (41.78%).

### 2.2 Research instruments

#### 2.2.1 Nature connectedness scale

Nature connectedness was measured using the Nature Connectedness Scale developed by Mayer and Frantz (2004), with the Chinese version revised by Li and Wu (2016). This scale consists of 14 items (2 reverse-scored), rated on a 5-point Likert scale. It is a unidimensional scale assessing the intensity of participants’ sense of connection with nature. Higher total scores indicate a stronger sense of nature connectedness. Previous research provides support for the scale’s use with primary school students (He et al., 2025). In this study, the scale demonstrated good internal consistency (Cronbach’s *α* coefficient = 0.77).

#### 2.2.2 Delaware social–emotional competence scale

Social–emotional competence (SEC) was assessed using the Delaware Social–Emotional Competence Scale developed by Mantz et al. (2018), translated and adapted into Chinese by Zhu (2016). The scale comprises 12 items (1 reverse-scored) measuring four dimensions: Responsible Decision-Making, Peer Relationships, Social Awareness, and Self-Management. Items were rated on a 4-point Likert scale. A total score was calculated by summing all item scores, with higher scores indicating greater SEC. The scale has been used with primary school students with acceptable psychometric properties (Wang, 2024). In this study, the scale demonstrated acceptable internal consistency (Cronbach’s *α* coefficient = 0.77).

#### 2.2.3 Basic empathy scale

Empathy was measured using the Basic Empathy Scale developed by Jolliffe and Farrington (2006), with the Chinese version revised by Li et al. (2011). This scale consists of 20 items (8 reverse-scored) assessing two dimensions: Cognitive Empathy and Affective Empathy. Items were rated on a 5-point Likert scale. A total empathy score was calculated by summing all item scores, with higher scores indicating higher levels of empathy. The scale has demonstrated acceptable psychometric characteristics for use with primary school students (Chen, 2024). In this study, the scale demonstrated acceptable internal consistency (Cronbach’s α coefficient = 0.72).

### 2.3 Statistical analysis

Data processing and analysis were conducted using SPSS 25.0. The mediation effect was tested using the Bootstrap method (Model 4) within Hayes’ PROCESS macro program.

The analytical procedure included:

1. Common Method Bias Check: Harman’s single-factor test was performed.
2. Descriptive Statistics and Correlations: Descriptive statistics (means, standard deviations) were calculated for all variables. Pearson correlation analysis was conducted to examine bivariate relationships.
3. Group Differences: Independent samples t-tests were used to examine gender and school differences in nature connectedness, empathy, and SEC. One-way analysis of variance (ANOVA) was used to examine grade-level differences (Grade 4 vs. 5 vs. 6) on these variables.
4. Mediation Analysis: Hayes’ PROCESS Macro (Model 4) was employed to test the mediating role of empathy in the relationship between nature connectedness and SEC. This study considered gender, grade level, and school as potential covariates. The decision rule was to retain in the final mediation model only those demographic factors that demonstrated significant associations with the core study variables in preliminary group comparisons. The analysis used a 95% confidence level with 5,000 bootstrap resamples. Mediation effects were considered statistically significant if the bias-corrected bootstrap confidence intervals did not include zero.

## 3 Results

### 3.1 Common method bias test

Given the use of self-reported data, common method bias (CMB) was assessed using Harman’s single-factor test. The analysis identified 13 factors with eigenvalues greater than 1. The first factor accounted for 14.60% of the total variance, which is below the critical threshold of 40%. This indicates that no significant common method bias was present in the study data (Podsakoff et al., 2003).

### 3.2 Descriptive statistics and correlation analysis

Table 1 presents the descriptive statistics and Pearson correlation coefficients among the key variables. Upper-grade Yi pupils reported a mean nature connectedness score of 44.65 (SD = 9.35), a mean SEC score of 35.36 (SD = 7.19), and a mean empathy score of 64.36 (SD = 9.74). Nature connectedness showed a significant positive correlation with SEC (*r* = 0.30, *p* < 0.01). Nature connectedness also showed a significant positive correlation with empathy (*r* = 0.22, *p* < 0.01). Empathy was significantly positively correlated with SEC (*r* = 0.34, *p* < 0.01).

**Table 1:** Descriptive statistics and correlations (*N* = 647).

Variable	M	SD	1	2	3	4	5	6	7	8	9
Nature connectedness	44.65	9.35	1								
Social–emotional competence	35.36	7.19	0.30**	1							
Responsible decision-making	9.17	1.98	0.22**	0.76**	1						
Peer relationships	8.62	2.38	0.27**	0.84**	0.53**	1					
Social awareness	8.96	2.28	0.26**	0.83**	0.50**	0.60**	1				
Self-management	8.62	2.20	0.22**	0.81**	0.49**	0.57**	0.59**	1			
Empathy	64.36	9.75	0.22**	0.34**	0.25**	0.28**	0.23**	0.33**	1		
Cognitive empathy	29.60	5.25	0.21**	0.38**	0.26**	0.32**	0.30**	0.36**	0.79**	1	
Affective empathy	34.76	6.50	0.16**	0.19**	0.17**	0.16**	0.11**	0.20**	0.87**	0.37**	1

**p < 0.01.

Correlation analysis revealed that although nature connectedness showed significant positive correlations with all subdimensions of social–emotional competence (SEC; *r* = 0.22–0.27, *p* < 0.01), the effect sizes varied. The correlations were slightly stronger with peer relationships (*r* = 0.27) and social awareness (*r* = 0.26) than with responsible decision-making (*r* = 0.22) and self-management (*r* = 0.22). Similarly, the correlations between empathy and the SEC subdimensions also showed a gradient pattern (cognitive empathy *r* = 0.30–0.38; affective empathy *r* = 0.11–0.20), suggesting that different subdimensions may function through distinct psychological mechanisms.

### 3.3 Group differences analysis

Gender Differences: Independent samples t-tests revealed significant gender differences, girls reported higher scores than boys on all three measures (Table 2).

**Table 2:** Differences in nature connectedness, empathy, and social–emotional competence by gender (*N* = 647).

Variable	Gender	N	M ± SD	*t*
Nature connectedness	Male	347	43.33 ± 9.26	−3.90***
	Female	300	46.18 ± 9.23	
Empathy	Male	347	62.72 ± 9.33	−4.70***
	Female	300	66.27 ± 9.8	
Social–emotional competence	Male	347	34.18 ± 7.59	−4.60***
	Female	300	36.72 ± 6.45	

***p < 0.001.

Nature connectedness: Girls (M = 46.18, SD = 9.23) vs. boys (M = 43.33, SD = 9.26), *t* = −3.90, *p* < 0.001.

Empathy: Girls (M = 66.27, SD = 9.8) vs. boys (M = 62.72, SD = 9.33), *t* = −4.70, *p* < 0.001.

SEC: Girls (M = 36.72, SD = 6.45) vs. boys (M = 34.18, SD = 7.59), *t* = −4.60, *p* < 0.001.

Grade Differences: One-way ANOVA results (Table 3) indicated no significant differences in nature connectedness (*F*(2,644) = 0.38, *p* = 0.68), empathy (F(2,644) = 0.89, *p* = 0.41), or SEC (F(2,644) = 0.21, *p* = 0.81) across grades 4, 5, and 6.

**Table 3:** Differences in nature connectedness, empathy, and social–emotional competence by grade (*N* = 647).

Variable	Grade	N	M ± SD	*F*	*p*
Nature connectedness	4	270	44.28 ± 8.10	0.38	0.68
	5	138	44.85 ± 10.57		
	6	239	44.97 ± 9.93		
Empathy	4	270	64.41 ± 9.09	0.89	0.41
	5	138	63.45 ± 9.57		
	6	239	64.84 ± 10.54		
Social–emotional competence	4	270	35.14 ± 6.14	0.21	0.81
	5	138	35.50 ± 6.71		
	6	239	35.52 ± 8.47		

School Differences: Independent samples t-tests were conducted to examine potential differences between the two participating schools on the key variables. As shown in Table 4, no significant differences were found in nature connectedness (*t* = −1.85, *p* = 0.07), empathy (*t* = −0.72, *p* = 0.47), or social–emotional competence (*t* = 1.77, *p* = 0.08).

**Table 4:** Differences in nature connectedness, empathy, and social–emotional competence by school (*N* = 647).

Variable	School	N	M ± SD	*t*	*p*
Nature connectedness	A	312	43.68 ± 8.91	−1.85	0.07
	B	335	45.56 ± 9.67		
Empathy	A	312	64.10 ± 9.42	−0.72	0.47
	B	335	64.61 ± 10.04		
Social–emotional competence	A	312	35.84 ± 6.31	1.77	0.08
	B	335	34.91 ± 7.90		

School names are anonymized as A and B.

### 3.4 Mediation analysis

Mediation analysis was conducted using Hayes’ PROCESS macro (Model 4) with 5,000 bootstrap samples. Gender was included as a covariate. Nature connectedness was the independent variable, SEC the dependent variable, and empathy the mediator.

Table 4 presents the standardized regression coefficients (*β*). The total effect of nature connectedness on SEC was significant (*β* = 0.28, *t* = 7.45, *p* < 0.001), supporting Hypothesis 1. Nature connectedness significantly predicted empathy (*β* = 0.20, *t* = 5.21, *p* < 0.001), supporting Hypothesis 2. Empathy significantly predicted SEC (*β* = 0.27, *t* = 7.20, *p* < 0.001), supporting Hypothesis 3.

When empathy was included in the model, the direct effect of nature connectedness on SEC remained significant but reduced (*β* = 0.23, *t* = 6.14, *p* < 0.001), indicating partial mediation. Gender also had significant direct and indirect effects via empathy. The model explained significant variance in SEC (*R^2^* = 0.17).

**Figure 2:**
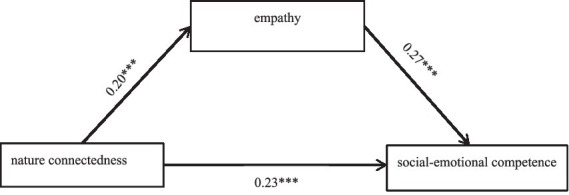
Mediation model (***p < 0.001).

Bootstrap analysis confirmed the significance of the indirect effect (Table 5; Figure 2). The total effect of nature connectedness on SEC was significant (*β* = 0.28, 95% CI [0.16, 0.27]). The direct effect remained significant (*β* = 0.23, 95% CI [0.12, 0.23]), accounting for 81.02% of the total effect. The indirect effect via empathy was significant (*β* = 0.04, 95% CI [0.02, 0.07]), accounting for 18.98% of the total effect. This supports Hypothesis 4, indicating empathy partially mediates the relationship between nature connectedness and SEC. The path diagram illustrating the final mediation model is presented in Figure 2.

**Table 5:** Regression analysis of variables in the mediation model (*N* = 647).

Predictor	SEC		empathy		SEC	
	*β*	*t*	*β*	*t*	*β*	*t*
Nature connectedness	0.28	7.45***	0.20	5.21***	0.23	6.14***
Gender	0.13	3.54***	0.15	3.95***	0.09	2.53*
Empathy					0.27	7.20***
*R* ^2^	0.11		0.07		0.17	
*F*	38.96***		25.06***		45.32***	

*p < 0.05, ***p < 0.01, ***p < 0.001; Coefficients are standardized (β).

Furthermore, to test the improvement in model fit after including the mediator, a hierarchical regression analysis was conducted. The results showed that after controlling for gender, adding empathy to the model with nature connectedness as the predictor variable and social–emotional competence as the outcome variable significantly increased the explained variance by 6% (ΔR^2^ = 0.06, *F*(1, 643) = 46.48, *p* < 0.001; Table 6). This result further supports the mediating role of empathy.

**Table 6:** Bootstrap effects test (*N* = 647, Bootstrap = 5,000).

Effect type	Path	Effect (*β*)	*SE*	95% CI lower	95% CI upper	Effect proportion
Total effect	Nature connectedness → SEC	0.28	0.03	0.16	0.27	100%
Direct effect	Nature connectedness → SEC	0.23	0.03	0.12	0.23	81.02%
Indirect effect	Nature connectedness → empathy → SEC	0.04	0.01	0.02	0.07	18.98%

## 4 Discussion

This study explored the relationship between nature connectedness and social–emotional competence (SEC) and the mediating role of empathy among upper-grade Yi ethnic minority pupils in rural Southwest China. The results revealed that nature connectedness significantly predicted SEC, and empathy partially mediated this relationship. These findings not only provide support for the applicability of existing theories within a culturally diverse context but also offer a novel explanatory framework for the psychosocial development of marginalized ethnic minority children.

### 4.1 The positive predictive relationship between nature connectedness and social–emotional competence

This study first identified a significant positive correlation between nature connectedness and children’s SEC (*r* = 0.30, *p* < 0.01), which was further supported by regression analysis demonstrating that nature connectedness significantly predicted SEC (*β* = 0.28, *p* < 0.001). This finding is consistent with previous research, aligning with the theoretical view that nature connectedness promotes children’s psychosocial development (Nagi et al., 2025; Feraco et al., 2025). Nature connectedness, enhancing children’s sense of belonging and emotional identification with the natural environment, may indirectly strengthen SEC through three potential mechanisms.

First, consistent with Attention Restoration Theory (ART; Kaplan, 1995) introduced earlier, prolonged exposure to natural environments may improve children’s attentional resource allocation and emotion regulation capacity, laying the foundation for social learning. Empirical research by Kuo et al. (2019) indicated that nature exposure significantly enhanced children’s self-control and social judgment. Second, natural environments often serve as settings for collective activities (e.g., herding, foraging, outdoor play), providing increased opportunities for social interaction and enhancing cooperation and communication skills (Kiviranta et al., 2023). Third, the emotional projection children form toward natural life forms (e.g., animals, plants) may transfer to the ability to recognize emotional cues and respond empathetically in interpersonal interactions, thereby strengthening core dimensions of SEC such as social awareness and emotion management (Johnstone et al., 2022).

Within the cultural context of Yi children, nature connectedness carries deeper sociocultural significance. Yi traditional culture emphasizes a “human-nature-ancestral spirits” cosmology. This imbues children’s experiences of nature with sacredness and endows learning and interactions within natural settings with socio-emotional meaning (Ping, 2025). For instance, collective nature-related rituals during the Torch Festival are not only expressions of nature worship but also crucial opportunities for emotional expression, role identification, and the construction of group belonging (Zhong, 2022). Thus, within this cultural milieu, nature connectedness functions not merely as a personal attitude or cognitive link to nature but as a bridge facilitating social learning and emotional socialization through nature.

### 4.2 The mediating role of empathy

The study further revealed that empathy partially mediated the relationship between nature connectedness and SEC (indirect effect = 0.04, proportion 18.98, 95% CI [0.02, 0.07]). This finding deepens our understanding of the mechanism linking nature connectedness and SEC, suggesting that children develop sensitivity and understanding toward “others” through nature experiences, thereby enhancing their empathy and social adaptability in interpersonal contexts. This pathway is consistent with the “Nature-Empathy-Psychological Trait” sequential model proposed by Fido and Richardson (2019).

How might nature experiences foster empathy? Existing research suggests that the neural mechanisms underlying affective resonance with natural life forms (e.g., injured animals, wilting plants) overlap with those involved in emotional responses to human “others” (Lengieza and Swim, 2021). Summers et al. (2019) further indicated that greater emotional investment in nature correlated with higher empathy scores in response to scenes of human suffering. The process through which nature experiences may foster empathy can be understood at a psychological level. Engaging with natural environments and non-human life likely requires and nurtures a general capacity for emotional attention and perspective-taking. This practiced sensitivity to the states of other living beings (e.g., caring for an injured animal) may then generalize to interpersonal contexts, forming a foundational skill for social–emotional competence. Future research employing neuroscientific methods could explore whether such behavioral changes are accompanied by specific patterns of neural plasticity (Steininger et al., 2025).

In this study, Yi children’s empathic experiences with nature may stem from embodied ecocultural practices in daily life, such as learning medicinal plant knowledge from elders, caring for young animals during herding, and performing ritualistic roles in nature-themed seasonal festivals. These activities provide opportunities to observe the states of “non-human life” and activate psychological pathways for recognizing emotional cues and constructing empathetic feelings. As Decety and Cowell (2014) emphasized, empathy is a capacity highly dependent on social experience and profoundly shaped by cultural structures.

### 4.3 Cultural interpretation of gender differences

The study also found that girls scored significantly higher than boys on nature connectedness, Empathy, and SEC (all *p* < 0.001). This pattern has been observed in some international studies, suggesting girls may be more inclined toward emotionally processing natural stimuli and establishing a sense of belonging with nature and cross-species empathy (Arnocky and Stroink, 2010; Christov-Moore et al., 2014). Gender role differentiation within cultural socialization processes may reinforce this trend: in traditional Yi communities, female children often participate more frequently in domestic tasks closely intertwined with nature (e.g., herb gathering, planting, livestock care) and nurturing responsibilities. These activities may strengthen their experiential understanding of the commonality between natural and human life, thereby promoting their SEC development.

From a socialization theory perspective, females are often encouraged to develop emotional expressiveness and social understanding skills, potentially leading to higher performance in SEC dimensions such as social awareness and self-management (Eisenberg et al., 2015). The current findings support this view and suggest the need for gender sensitivity in educational interventions. Particularly when designing Social–Emotional Learning (SEL) activities in natural settings, it may be beneficial to create more challenging and interactive natural tasks for boys (e.g., collaborative exploration, biodiversity monitoring) to stimulate their natural affection and empathic experiences.

### 4.4 Theoretical contributions and interdisciplinary significance

The primary theoretical contribution of this study lies in its empirical examination, within a Chinese Southwest ethnic minority child population, of the mechanism pathway. This provides a significant example of integrating perspectives from developmental psychology, environmental psychology, and educational psychology. Compared to previous research predominantly focused on urban middle-class children in Western contexts, this study extends inquiry beyond cultural centrism and advances the field of nature-psychology research among Chinese ethnic minority children.

More importantly, this study reconstructs the connotation of nature connectedness from a culturally embedded perspective: for Yi children, nature is not merely an external environment but a carrier of life ethics, historical narratives, and social identity. Consequently, connection with nature transcends environmental attitudes or esthetic preferences; it pertains to the cognitive and emotional integration of the “self-other-ethnic group” dimensions. This perspective contributes to expanding developmental psychology’s understanding of the “culture-psychological development” interaction pathways.

### 4.5 Practical implications

The findings hold significant implications for education in rural ethnic regions. First, schools and communities can systematically enhance children’s sense of nature connectedness through nature-based education initiatives (e.g., establishing nature classrooms, organizing ecological festivals). Second, empathy training should be systematically integrated into SEL curricula, utilizing methods like nature stories, role-playing, and situational dramas to enhance students’ understanding and emotional responsiveness toward both natural and human “others.” Third, culturally integrated SEL models should be explored. Leveraging Yi cultural elements—such as myths, festivals, and rituals—as concrete educational contexts can achieve the dual goals of cultural preservation and competency cultivation.

Furthermore, education policymakers should recognize the unique value of natural environments for child development in ethnic regions. Within China’s rural revitalization and “Double Reduction” policy context, integrating natural spaces with SEL may become a vital pathway for promoting educational equity and ethnic unity.

### 4.6 Limitations and future directions

Despite its theoretical and practical value, this study has several limitations. First, the cross-sectional design, while allowing us to test the statistical plausibility of a proposed mediation model consistent with causal theory, limits the strength of causal inferences that can be drawn. The observed associations and mediation effects are consistent with the hypothesized directional pathways, but alternative explanations, such as reverse or bidirectional causality, cannot be ruled out. For instance, children with higher SEC might seek out or perceive more connection with nature. Future research should employ longitudinal tracking or experimental intervention designs to further examine the causal pathways linking nature connectedness to SEC. Second, reliance on self-report measures carries risks of social desirability bias. Triangulation using teacher evaluations, peer nominations, or behavioral observations is recommended. Third, the current study focused primarily on empathy. Future research should explore more complex socio-cognitive processes (e.g., perspective-taking, moral judgment) as potential bridging mechanisms between nature experiences and SEC. Fourth, the sample was confined to Yi communities, limiting generalizability to other ethnic minorities or multicultural contexts. Comparative studies across diverse regions and ethnic groups are encouraged. Finally, this study did not conduct an in-depth examination of the mediating effects for the subdimensions of empathy and SEC. In particular, the correlations between cognitive empathy and the various SEC dimensions (*r* = 0.30–0.38) were generally higher than those for affective empathy (*r* = 0.11–0.20). This suggests that nature connectedness may influence different aspects of SEC through distinct empathic mechanisms. For example, nature connectedness might primarily promote social awareness by enhancing cognitive empathy (the ability to understand others’ perspectives), while strengthening peer relationships through affective empathy (the ability to share others’ emotions). These nuanced mechanistic differences warrant further exploration in future research with larger samples and more complex designs.

## 5 Conclusion

This study, conducted among upper-grade Yi pupils in rural Southwest China, revealed a significant positive predictive relationship between nature connectedness and social–emotional competence (SEC), with empathy acting as a partial mediator. Specifically, children with stronger nature connectedness exhibited higher levels of SEC, and this promotive association was partially achieved through the enhancement of empathic capacity.

This finding not only enriches the cultural and developmental perspective of nature connectedness research but also provides preliminary evidence for the mechanistic role of the natural environment in the socio-emotional development of rural ethnic minority children. The sense of belonging with nature, cultivated by Yi children through daily natural practices, helps foster empathic understanding of nature and “others.” This empathic capacity subsequently transfers to broader interpersonal contexts, thereby promoting overall social adaptation and mental health development.

This study emphasizes that efforts to promote child development in ethnic regions should fully leverage ecological and cultural resources. Constructing social–emotional education pathways that use nature as a medium and empathy as a hub is crucial. Future research should expand methodological designs, explore more complex psychological mechanisms, and conduct cross-cultural comparisons to further enrich the theoretical construction and practical intervention systems of the “nature-psycho-social” development pathway.

## Data Availability

The raw data supporting the conclusions of this article will be made available by the authors, without undue reservation.
